# Exploration of reasons for primary care testing (the Why Test study): a UK-wide audit using the Primary care Academic CollaboraTive

**DOI:** 10.3399/BJGP.2023.0191

**Published:** 2023-10-03

**Authors:** Jessica Watson, Alexander Burrell, Polly Duncan, Ian Bennett-Britton, Sam Hodgson, Samuel WD Merriel, Salman Waqar, Penny F Whiting, The Primary care Academic CollaboraTive

**Affiliations:** National Institute for Health and Care Research doctoral research fellow;; National Institute for Health and Care Research doctoral research fellow;; National Institute for Health and Care Research doctoral research fellow;; Centre for Academic Primary Care, University of Bristol, Bristol.; Wolfson Institute of Population Health, Queen Mary University of London, London.; Exeter Collaboration for Academic Primary Care, Exeter Medical School, University of Exeter, Exeter; Centre for Primary Care & Health Services Research, University of Manchester, Manchester.; Department of Primary Care and Public Health, Imperial College London, London.; Population Health Sciences, Bristol Medical School, University of Bristol, Bristol.

**Keywords:** blood tests, collaborative research, clinical decision-making, diagnosis, overtesting, primary health care

## Abstract

**Background:**

Rates of blood testing have increased over the past two decades. Reasons for testing cannot easily be extracted from electronic health record databases.

**Aim:**

To explore who requests blood tests and why, and what the outcomes of testing are in UK primary care.

**Design and setting:**

A retrospective audit of electronic health records in general practices in England, Wales, Scotland, and Northern Ireland was undertaken.

**Method:**

Fifty-seven clinicians from the Primary care Academic CollaboraTive (PACT) each reviewed the electronic health records of 50 patients who had blood tests in April 2021. Anonymised data were extracted including patient characteristics, who requested the tests, reasons for testing, test results, and outcomes of testing.

**Results:**

Data were collected from 2572 patients across 57 GP practices. The commonest reasons for testing in primary care were investigation of symptoms (43.2%), monitoring of existing disease (30.1%), monitoring of existing medications (10.1%), and follow up of previous abnormalities (6.8%); patient requested testing was rare in this study (1.5%). Abnormal and borderline results were common, with 26.6% of patients having completely normal test results. Around one-quarter of tests were thought to be partially or fully unnecessary when reviewed retrospectively by a clinical colleague. Overall, 6.2% of tests in primary care led to a new diagnosis or confirmation of a diagnosis.

**Conclusion:**

The utilisation of a national collaborative model (PACT) has enabled a unique exploration of the rationale and outcomes of blood testing in primary care, highlighting areas for future research and optimisation.

## INTRODUCTION

Routine data from primary care electronic health records has demonstrated a more than threefold increase in the use of laboratory tests in UK primary care between 2000 and 2016,^1^ with significant variation in testing rates between GP practices.^2^ This rise in testing has taken place in the context of significant uncertainty and lack of evidence to determine which tests are ‘necessary’, with guidelines for chronic disease monitoring mostly based on expert opinion.^3^

Estimates have suggested that 25% of primary care laboratory tests might be ‘unnecessary’,^4^ with research demonstrating unwarranted variation^5^ and overuse of specific tests including thyroid function tests, liver function tests, prostate- specific antigen tests, and vitamin D tests.^6^ This may lead to further blood tests, imaging, appointments, and referrals, a process sometimes referred to as the ‘cascade effect’.^7^ The concept of the cascade effect has been around for over 30 years^8^ but is rarely measured,^9^ and the overall frequency and implications of cascade testing on primary care workload is unknown.

Reduction in unwarranted variation in testing rates has been frequently cited as an aim,^10^ particularly in the current UK context of rising workload,^11^ a primary care workforce crisis, and concerns about socioeconomic inequalities in health. A prerequisite to achieving this aim is to first understand the rationale for blood testing in primary care, and the outcomes of testing. This information cannot be obtained easily from current electronic health record data.

The Primary care Academic CollaboraTive (PACT) is a new UK-wide network of primary care health professionals from England, Wales, Scotland, and Northern Ireland, who collectively take part in primary care research and quality improvement projects that seek to improve patient care.^12^ The aim of this study was to use the PACT collaborative research model to explore who requests blood tests and why, and what the outcomes of testing are in UK primary care.

## METHOD

### Recruitment and sampling

Full details of the methods for this study have been published previously.^13^ Data were collected by primary care clinicians, including GP registrars, GPs, and allied health professionals (hereinafter ‘PACT members’), by extracting data on recent blood tests undertaken in the GP practice where they were working. Information about the study was disseminated via the PACT newsletter, social media, Clinical Research Networks, GP registrar newsletters, and the newsletter of a national GP leadership programme (‘Next Generation GP’). PACT members completed an online expression of interest and consent form. A GP partner or practice manager was then required to complete a practice agreement form.

**Table:** How this fits in

Previous research has shown a more than threefold increase in the use of laboratory tests in UK primary care between 2000 and 2015, with significant variation in testing rates between GP practices. In this study, around one-quarter of tests were thought to be partially or fully unnecessary when reviewed retrospectively by another clinician. Around half of tests (48.8%) did not lead to any change in management or reassurance; 13.4% led to further blood tests or repeat blood tests and 2.7% led to further radiology tests. Overall, 6.2% of tests in primary care led to a new diagnosis or confirmation of diagnosis. This has important implications for how primary care clinicians should talk to patients about blood tests, ensuring that patients have a better understanding and more realistic expectations of the role of blood tests in their care.

Purposive sampling was used to recruit the first seven pilot GP practices, include a range of PACT team members and a range of electronic health records systems (EMIS, SystmOne, and Vision), and identify any problems with data-collection tools before the wider rollout. All PACT members who expressed an interest were invited to take part, with an aim of recruiting at least 50 practices.

### Training

PACT members were required to watch two short training videos and code three fictitious clinical cases using a computerised database (REDCap) before commencing data collection (see Supplementary Box S1).

A pass mark of >70% for each of the three test cases was set; all participants exceeded this on the first attempt for each of the three training cases, with mean scores of 95% for case 1 (range 73%–100%), 94% for case 2 (range 80%–100%), and 94% for case 3 (range 73%–100%). Supplementary Table S1 shows training case scores for GPs, compared with GP registrars and allied health professionals.

### Data collection

The research team provided PACT members with an automated search to identify a random sample of eligible participants from their GP practices’ electronic health records system. Eligible patients were anyone aged ≥18 years having a blood test in primary care during April 2021. This period was chosen pragmatically to capture usual practice following the early waves of the COVID- 19 pandemic, and to allow sufficient time for follow up before issues with primary care blood bottle shortages in the UK during August 2021. Pregnant women were excluded manually by the PACT members because of biochemical differences in reference ranges for routine bloods.

Each PACT member reviewed the notes of 50 patients and manually extracted anonymised data into a REDCap database on: patient demographics (age and gender), type of clinician who requested testing, primary reasons for testing (plus optional secondary reason for testing), symptoms triggering testing, test results, and the outcomes of testing (new diagnosis, medication change, lifestyle recommendation, referral, hospital admission, further tests, reassurance, or ‘none of the above’). Symptoms triggering testing were subcategorised using the International Classification for Primary Care (ICPC2).^14^

Separate components of a test (for example, individual analytes of a full blood count) were grouped and counted as one test. For each test the results were categorised by PACT members into normal (‘all test results are within the laboratory specified reference ranges’), borderline (‘one or more tests are very slightly outside of the laboratory specified reference range’), or abnormal (‘one or more tests are definitely outside of the normal range’).

Categorical and free-text data were collected on how test results were coded, actioned, and communicated, which will be reported separately.^15^ The final question PACT members were asked to respond to for each patient was: ‘In your clinical opinion, were the tests necessary?’. This could be categorised into a) yes, all tests were necessary; b) some tests were necessary, but not all; or c) no tests were necessary. It was emphasised that this question relies on clinical judgement and these findings should be seen as exploratory in nature.

### Analysis

Results were analysed using simple descriptive statistics. Logistic regression analysis was used to estimate the association between the frequency of abnormal results and patient age group, gender, the reason for testing, and the type of clinician who requested the test. Results were presented using odds ratios (ORs) and corresponding 95% confidence intervals (CIs) to quantify the strength and direction of the association between each independent variable and the frequency of abnormal blood test results. All statistical analyses were performed using Stata (version 17).

## RESULTS

**Figure 1.**
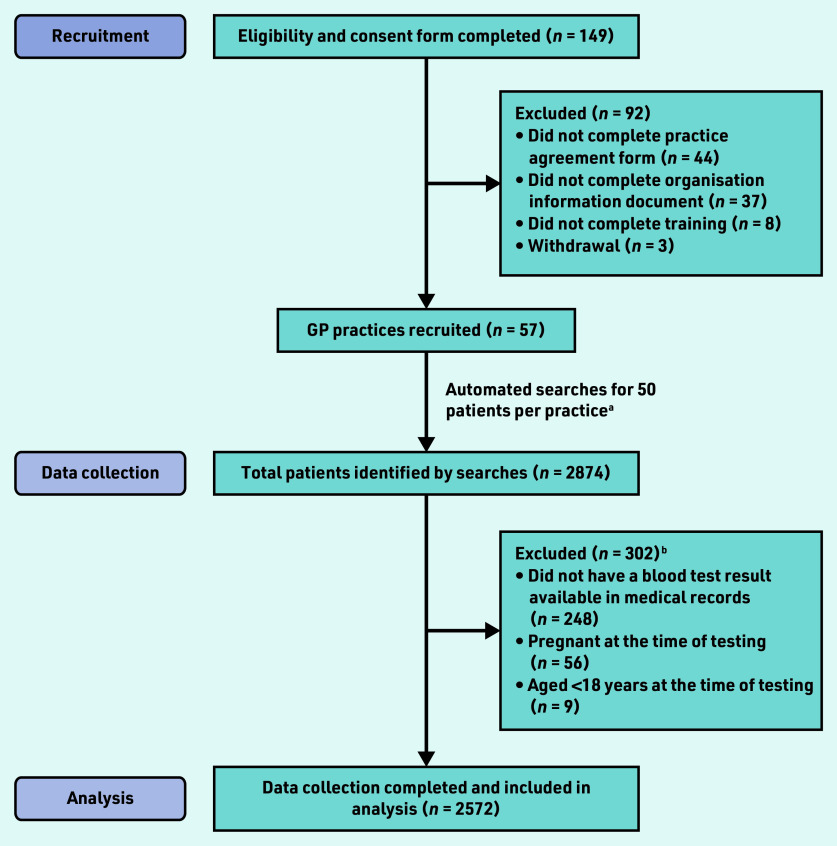
*Participant flowchart. ^a^Total figure slightly higher than expected because: two practices had problems with the automated searches identifying a large number of ineligible patients and therefore ‘topped up’ their dataset; five practices completed 51 and one practice completed 53 data-collection forms without explanation; and for three small practices the search returned <50 eligible patients. ^b^More than one reason for exclusion could be selected.*

Eligibility and consent forms were received from 149 PACT members, from which a total of 57 PACT members (from 57 GP practices) were recruited; 92 PACT members who expressed an initial interest did not complete the relevant study documentation required for participation or withdrew from the study (Figure 1).

Recruited practices came from England (*n* = 46), Scotland (*n* = 4), Wales (*n* = 5), and Northern Ireland (*n* = 2); demographics of participating practices are shown in Table 1. The majority of participating practices had list sizes between 5000 and 15 000 (38.6% 5001–10 000; 33.3% 10 001–15 000), this compares with an average practice list size of 9544 in England.^16^

**Table 1.** Demographics of practices and patients

**Characteristic**	***n* (%)**
**Practice demographics (*n* = 57)**	
Practice size	
≤5000	7 (12.3)
5001–10 000	22 (38.6)
10 001–15 000	19 (33.3)
15 001–20 000	7 (12.3)
20 001–25 000	0
25 001–30 000	0
>30 000	2 (3.5)

**Region**	
North East and North Cumbria	5 (8.8)
North West Coast	2 (3.5)
Yorkshire and Humber	3 (5.3)
Greater Manchester	4 (7.0)
East Midlands	6 (10.5)
West Midlands	3 (5.3)
West of England	1 (1.8)
Thames Valley and South Midlands	2 (3.5)
Eastern	5 (8.8)
Kent, Surrey and Sussex	1 (1.8)
Wessex	1 (1.8)
South West Peninsula	7 (12.3)
North Thames	3 (5.3)
South London	2 (3.5)
North West London	1 (1.8)
Wales	5 (8.8)
Scotland	4 (7.0)
Northern Ireland	2 (3.5)

**Practice population**	
Urban	31 (54.4)
Rural	13 (22.8)
Suburban	13 (22.8)

**Practice level IMD^a^**	
1 (most deprived)	11 (19.3)
2	5 (8.8)
3	8 (14.0)
4	4 (7.0)
5	6 (10.5)
6	7 (12.3)
7	4 (7.0)
8	4 (7.0)
9	4 (7.0)
10 (least deprived)	4 (7.0)

**Patient demographics (*n* = 2572)**	
Patient age, years	
18–29	238 (9.3)
30–39	322 (12.5)
40–49	360 (14.0)
50–59	449 (17.5)
60–69	505 (19.6)
70–79	442 (17.2)
≥80	256 (10.0)

**Gender**	
Male	1075 (41.8)
Female	1495 (58.1)
Other	2 (0.08)

a *Calculated using Fingertips data for practices in England^16^ and using GP practice postcode IMD for devolved nations: Scotland,^17^ Wales,^18^ Northern Ireland.^19^ IMD = Index of Multiple Deprivation.*

Practices were recruited from all regions of the UK, with slightly higher numbers in the South West Peninsula (12.3%) and East Midlands (10.5%). Practice-level Index of Multiple Deprivation data show a higher number of practices were recruited in more deprived than less deprived areas, including 19.3% in the most deprived areas, in keeping with PACT aims of broadening participating in research.

Participating PACT members included GP registrars (*n* = 32), First5 GPs (within 5 years of qualifying as a GP; *n* = 5), post- First5 GPs (*n* = 15), nurse practitioners (*n* = 2), practice pharmacists (*n* = 2), and a physician associate (*n* = 1) (data not shown).

After exclusions, data on a total of 2572 patients were included in the analysis (Figure 1). Age and gender of included patients are shown in Table 1. The cohort was 58.1% female, with the majority of tested patients between the ages of 50 and 79 years, in keeping with previous research exploring the demographics of primary care testing.^1^

Table 2 shows the tests performed and frequency of borderline and abnormal results. The most commonly performed test was urea and electrolytes (U&Es) followed by full blood count (FBC), liver function tests (LFTs), and glycated haemoglobin (HbA1c). The tests that most commonly led to borderline or abnormal results were vitamin D, FBC, and lipid profile; with FBC leading to the largest proportion of borderline results (34.4%).

**Table 2.** Tests performed and frequency of borderline and abnormal results^a^

**Name of test**	**Normal results, *n* (%)**	**Borderline results, *n* (%)^a^**	**Abnormal results, *n* (%)^a^**	**Total tests, *n***
U&Es (with/without potassium)	1411 (72.6)	295 (15.2)	238 (12.2)	1944
Full blood count	893 (49.8)	618 (34.4)	283 (15.8)	1794
Liver function tests	1184 (75.8)	214 (13.7)	164 (10.5)	1562
HbA1c	850 (69.9)	127 (10.4)	239 (19.7)	1216
Thyroid function tests	904 (87.8)	38 (3.7)	88 (8.5)	1030
Lipid profile	489 (53.4)	195 (21.3)	231 (25.2)	915
Bone profile	414 (87.9)	46 (9.8)	11 (2.3)	471
Haematinics	440 (64.7)	79 (11.6)	161 (23.7)	680
Vitamin D	87 (44.8)	22 (11.3)	85 (43.8)	194
Glucose	82 (76.6)	8 (7.5)	17 (15.9)	107
Coeliac screen	101 (99.0)	0 (0)	1 (1.0)	102
Other^b^	554 (78.5)	40 (5.7)	112 (15.9)	706

a *‘Borderline results’ defined as ‘very slightly outside of the laboratory specified reference range’; ‘abnormal’ defined as ‘definitely outside of the normal range’.*

b *Tests with* n*<100 merged into ‘other’ category. HbA1c = glycated haemoglobin. U&E = urea and electrolytes.*

The mean number of tests done simultaneously was 4.5 tests (standard deviation [SD] 2.4) per patient (counting FBC, U&E, and LFTs as a single ‘test’, rather than counting each analyte separately) (Table 3). If all simultaneous tests performed on an individual patient were considered, ≥1 of these tests were coded as ‘abnormal’ in 1176 (45.7%) patients; hereinafter this is referred to as ‘abnormal’ (data not shown). In 712 (27.7%) ≥1 tests were coded as ‘borderline’ with no ‘abnormal’ results; hereinafter ‘borderline’. In 684 (26.6%) patients, all tests were within the laboratory specified reference range; hereinafter ‘normal’.

**Table 3.** Primary reason for testing and frequency of abnormal results (*n* = 2572)

**Primary reason for testing**	**Patients tested, *n* (%)**	**Number of tests per patient, mean (SD)**	**Frequency of abnormal results, %**	**OR (95% CI)^a^**
Monitoring of existing disease	773 (30.1)	4.3 (2.1)	56.4	Reference
Monitoring of existing medication	259 (10.1)	3.1 (1.8)	37.8	0.48 (0.36 to 0.64)
Starting new medication	43 (1.7)	2.4 (1.4)	25.6	0.26 (0.12 to 0.52)
Symptoms/diagnosis	1111 (43.2)	5.5 (2.4)	42.0	0.53 (0.41 to 0.67)
Screening	17 (0.7)	2.7 (2.1)	17.6	0.18 (0.05 to 0.64)
Patient request	39 (1.5)	4.3 (2.7)	33.3	0.40 (0.20 to 0.80)
Follow up/repeat of previous abnormal result	174 (6.8)	2.4 (2.0)	49.4	0.68 (0.47 to 0.98)
Unclear	106 (4.1)	4.6 (2.1)	40.6	0.50 (0.31 to 0.82)
Other	50 (1.9)	4.1 (2.3)	36.4	0.45 (0.23 to 0.86)
Total	2572 (100)	4.5 (2.4)	45.7	N/A

a *Proportion of abnormal tests, compared with reference group (monitoring tests), adjusted for patient age, gender, and type of clinician requesting the test. N/A = not applicable. OR = odds ratio. SD = standard deviation.*

Table 4 shows which member of the primary care team requested blood tests, the number of tests requested on average by each type of clinician, and proportion of tests that were abnormal. Tests were most commonly requested by GPs (47.0%). Logistic regression (adjusted for age, gender, and reason for testing) showed lower rates of abnormal test results for nurse practitioners (OR 0.54, 95% CI = 0.36 to 0.79, *P* = 0.002), tests requested according to practice protocols (OR 0.74, 95% CI = 0.57 to 0.98, *P* = 0.03), and tests requested by secondary care (OR 0.58, 95% CI = 0.40 to 0.85, *P* = 0.005) compared with tests requested by GPs.

**Table 4.** Member of the healthcare team made the clinical decision to request the blood test (*n* = 2572)

**Healthcare team member**	**Patients tested, *n* (%)**	**Number of tests per patient, mean (SD)**	**Frequency of abnormal results, %**	**OR of receiving an abnormal result (95% CI)^a^**	***P*-value**
GP	1210 (47.0)	4.76 (2.56)	47.1	Reference	Reference
GP registrar	190 (7.4)	4.97 (2.41)	37.9	0.78 (0.56 to 1.07)	0.120
Locum GP^b^	106 (4.1)	5.08 (2.50)	50.0	1.24 (0.83 to 1.86)	0.296
Nurse practitioner	131 (5.1)	4.53 (2.52)	32.8	0.54 (0.36 to 0.79)	0.002
Nurse	94 (3.7)	4.69 (2.76)	47.9	0.86 (0.55 to 1.34)	0.501
Healthcare assistant	43 (1.7)	4.74 (2.37)	46.5	0.66 (0.35 to 1.26)	0.207
Pharmacist	33 (1.3)	3.76 (2.12)	39.4	0.69 (0.33 to 1.45)	0.328
Paramedic	1 (0.04)	3 (0)	0.0	— ^c^	— ^c^
Physician associate	24 (0.9)	5.42 (2.84)	45.8	1.18 (0.52 to 2.70)	0.694
Secondary care request	139 (5.4)	3.1 (2.08)	38.1	0.58 (0.40 to 0.85)	0.005
Protocol	499 (19.4)	4.16 (1.87)	50.5	0.74 (0.57 to 0.98)	0.033
Unclear/other	102 (4.0)	3.89 (2.29)	44.0	0.87 (0.49 to 1.52)	0.622

a *Odds of receiving an abnormal test result by clinician group compared with GP testing (adjusted for age, gender, and reason for testing).*

b *Locum GP category was not available for the pilot practices (*n *= 7) so locums were included within the ‘GP’ category in the pilot practices.*

c *Unavailable due to low number of participating paramedics. OR = odds ratio. SD = standard deviation.*

Table 3 shows the primary reasons for testing, mean number of tests requested, and the frequencies of abnormal results. The commonest reason for testing was investigation of symptoms (43.2%), followed by monitoring of existing disease (30.1%), monitoring of existing medication (10.1%), and follow up/repeat of previous abnormal result (6.8%). Testing to investigate symptoms was associated with the largest number of simultaneous blood tests (mean 5.5 tests) followed by monitoring of existing disease (mean 4.3 tests). Starting new medications and follow up/repeat of previous abnormal result were associated with the smallest number of tests (mean 2.4 tests, respectively).

Monitoring of existing disease yielded the highest frequency of abnormal results (56.4%), followed by follow up/repeat of previous abnormal result (49.4%), and investigation of symptoms (42.0%) (Table 3). In patients having testing to investigate symptoms (*n* = 1111), the most frequently recorded symptoms were ‘general and unspecified’ (20.1%), followed by digestive symptoms (17.0%) and musculoskeletal symptoms (12.2%). For full details of symptoms triggering testing see Supplementary Table S2. Supplementary Table S3 shows both primary and secondary reasons for testing; secondary reasons were optional and were completed in 954 out of 2572 patients.

Table 5 shows the outcomes of blood testing. Overall, around half of tests (48.8%) led to no change in patient outcomes. The commonest outcome of testing was change in medication/new medication (15.9%) followed by further blood tests/repeat blood tests (13.4%). Overall, 6.2% of tests led to a new diagnosis or confirmation of a diagnosis. Supplementary Table S4 shows how the outcomes of testing varied according to the reason for testing. In the 1111 patients who were having tests for investigation of symptoms, 109 (9.8%) led to a new diagnosis or confirmation of a diagnosis, with 460 (41.4%) leading to no change in outcomes.

The final question in the study was ‘In your clinical opinion, were the tests necessary?’. Overall, in 1927 (74.9%) patients, all tests were felt to be necessary, in 538 (20.9%) patients, some tests were felt to be necessary but not all, while in 107 (4.2%) patients, no test was felt to be necessary. Supplementary Table S5 shows how the frequency of tests that were felt to be necessary varied, depending on the indication for testing.

**Table 5.** Outcomes of blood testing (*n* = 2572)

**Consequences of testing**	***n* (%)^a^**
New diagnosis/confirmation of diagnosis	159 (6.2)
Change in medication/new medication	409 (15.9)
Change in lifestyle recommended	222 (8.6)
Referral	190 (7.4)
Hospital admission	11 (0.4)
Further blood tests/repeat blood tests	345 (13.4)
Follow-on X-ray/radiology investigations	69 (2.7)
Reassurance of doctor/patient	194 (7.5)
None of the above^b^	1256 (48.8)
Unclear	129 (5.0)

a *Total >100% as >1 option could be chosen simultaneously.*

b *The category ‘none of the above’ was used to identify tests where no change in outcomes could be identified following testing; the authors avoided using the wording ‘no change in outcomes’ to reduce potential subjective interpretation of what could be defined as a ‘change in outcome’.*

## DISCUSSION

### Summary

The commonest reasons for testing in primary care were investigation of symptoms (43.2%), monitoring of existing disease (30.1%), and monitoring of existing medications (10.1%). Only around half of tests in primary care were requested by GPs, reflecting the multidisciplinary nature of UK primary care.

On average, 4.5 tests were requested simultaneously per patient, and abnormal and borderline results were common, with only 26.6% of patients having completely normal test results. Around one- quarter of tests were thought to be partially or fully unnecessary when reviewed retrospectively by another clinician. Overall, 6.2% of tests in primary care led to a new diagnosis or confirmation of diagnosis. Nearly half of tests (48.8%) did not lead to any change in management or reassurance. Tests actioned to monitor existing disease led to the highest frequency of abnormal results; this is to be expected given that patients with chronic conditions such as type 2 diabetes or chronic kidney disease would be expected to have abnormal tests as a result of their condition.

To the authors’ knowledge, this is the first study to demonstrate the potential of the PACT collaborative research model to conduct research in primary care, with a range of clinicians and practices participating from across the four nations of the UK.

### Strengths and limitations

This study demonstrates the benefits of the PACT model; clinicians were able to extract data from patient records that could not have been collected using routine data from electronic health records. In particular, it was possible to address questions that require clinical interpretation, such as ‘In your clinical opinion, were the tests necessary?’. Other audits, such as the national cancer diagnosis audit, have used similar methods successfully.^20^ The authors of the current study provided rigorous training and tested PACT members using exemplar clinical cases before data collection to improve reliability. However, by definition some questions were based on clinical opinion, and will therefore vary between clinicians; these should be interpreted as exploratory in nature. The aim was not to provide independent expert opinion on whether tests in primary care are necessary or not, but to explore the variation in decision making between clinicians and explore how perspectives on testing might change with the benefit of hindsight. Data were collected by PACT members who were clinicians in the participating practices. It is therefore possible that they may have been reviewing patients where they had ordered/acted on the results, which could influence how they answered questions on how ‘necessary’ the tests were.

The COVID-19 pandemic caused significant disruptions to primary care testing; the choice of sampling tests done in April 2021 was pragmatic and may not fully reflect ‘post-pandemic’ testing patterns. The aim was to make the study as inclusive as possible, inviting all practices that expressed an interest to participate. Despite this, a broad range of participating practices was achieved, with slightly higher rates of recruitment from the most deprived areas, in keeping with the PACT ethos of broadening participation in research.

### Comparison with existing literature

Previous estimates based on expert opinion have suggested that 25% of tests in primary care may not be fully necessary.^4^ A review of primary care studies found overtesting rates between 0.2% and 100%, using a range of definitions of ‘overtesting’ for different types of test.^21^ To the authors’ knowledge, this is the first study to quantify the overall proportion of unnecessary blood testing in primary care; the finding that 74.9% of tests were fully necessary is broadly in keeping with other studies.

Previous estimates have suggested that up to 50% of primary care testing may be for chronic disease monitoring;^4^ the figures in the current study of 30.1% for disease monitoring and 10.1% for medication monitoring are slightly lower than this but nonetheless represent a very significant proportion of primary care testing.

Although the concept of ‘cascade testing’^7^ has been around for many years, there is limited previous empirical research. One small observational study in the Netherlands (*n* = 256) found that GPs ordered further investigations in 17.3% of patients;^9^ this is in keeping with the current data that show repeat blood tests were requested in 13.4% of patients, and follow- on X-ray/radiology tests were requested in 2.7% of patients.

### Implications for research and practice

This study has shown a high frequency of borderline results, particularly for tests with multiple component analytes such as FBC. This is unlikely to surprise practising clinicians, but has important implications given the move to offer all patients in England access to their blood test results via the NHS App.^22^

Further research to explore how patients interpret these borderline results is needed; although a clinician might mark these as ‘satisfactory’ or ‘normal’, if patients see that their test results are outside the reference range it could potentially trigger alarm. This is backed up by data from the US; after implementation of online records access, one medical centre measured a doubling in the number of messages sent by patients within 6 h after patients reviewed a result.^23^ Although borderline tests are common incidental findings, it is important to recognise that they could also reflect early manifestations of underlying disease; for example, borderline thrombocytosis is associated with an increased risk of cancer.^24^

The finding that up to one-quarter of tests may be fully or partially unnecessary is particularly important given workload pressures in primary care.^11^ Further research is needed to develop objective measures of inappropriate testing, similar to the extensive work that has been undertaken to develop measures of potentially inappropriate prescribing.^25^ This could form the foundation for research to optimise the use of tests in primary care and reduce unwarranted variation.

Unsurprisingly, given the increasingly multidisciplinary nature of primary health care in the UK, the current study found tests were requested by the full range of multidisciplinary team members, with less than half of tests directly requested by GPs. This raises questions about how to ensure all healthcare professionals receive training in ordering and interpreting blood tests.

Qualitative studies have shown that patients tend to have high expectations of blood tests, hoping they will provide answers and solutions to their symptoms.^26^ However, this study showed that 6.2% of tests led to a new diagnosis or confirmation of a diagnosis; when tests were requested for symptoms this figure was 9.8%. This has important implications for how clinicians talk to patients about blood testing, ensuring that patients have a better understanding and realistic expectations of the role of blood tests in their care.

This study has demonstrated the potential of the PACT model for conducting research, opening up opportunities for further research using this collaborative model. A full evaluation of the PACT model is underway to inform the development of future collaborative research studies.

In conclusion, the utilisation of a national collaborative model (PACT) has enabled a unique exploration of the rationale and outcomes of blood testing in primary care, highlighting areas for future research and quality improvement to optimise the use of blood tests in primary care.

## Data

Data available from authors on reasonable request.
